# Finnish Version of the Eating Assessment Tool (F-EAT-10): A Valid and Reliable Patient-reported Outcome Measure for Dysphagia Evaluation

**DOI:** 10.1007/s00455-021-10362-9

**Published:** 2021-09-13

**Authors:** Pia Järvenpää, Jonna Kuuskoski, Petra Pietarinen, Mari Markkanen-Leppänen, Hanna Freiberg, Miia Ruuskanen, Jami Rekola, Taru Ilmarinen, Teemu J. Kinnari, Timo J. Autio, Elina Penttilä, Marika S. Muttilainen, Annika Laaksonen, Lotta Oksanen, Ahmed Geneid, Leena-Maija Aaltonen

**Affiliations:** 1 Department of Otorhinolaryngology – Head and Neck Surgery, Helsinki University Hospital and University of Helsinki, Helsinki, P.O. Box 263, FI-00029 Finland; 2 Department of Otorhinolaryngology – Head and Neck Surgery, Turku University Hospital and University of Turku, Turku, Finland; 3 Department of Otorhinolaryngology – Head and Neck Surgery, Oulu University Hospital, Oulu, Finland; 4 PEDEGO Research Unit, University of Oulu, Oulu, Finland; 5 Medical Research Center Oulu, Oulu, Finland; 6 Department of Otorhinolaryngology – Head and Neck Surgery, Kuopio University Hospital and University of Eastern Finland, Kuopio, Finland; 7 Department of Rehabilitation and Psychosocial Support, Tampere University Hospital and Faculty of Social Sciences, University of Tampere, Tampere, Finland; 8 Department of Otorhinolaryngology and Phoniatrics – Head and Neck Surgery, Helsinki University, Hospital and University of Helsinki, Helsinki, Finland

**Keywords:** Dysphagia, EAT-10, Patient-reported outcome measure, Dysphagia screening, Swallowing, Deglutition

## Abstract

Our aim was to validate a Finnish version of the Eating Assessment Tool (F-EAT-10) for clinical use and to test its reliability and validity in a multicenter nationwide study. Normative data were acquired from 180 non-dysphagic participants (median age 57.0 years, 62.2% female). Dysphagia patients (*n* = 117, median age 69.7 years, 53.0% female) referred to fiberoptic endoscopic evaluation of swallowing (FEES) completed F-EAT-10 before the examination and after 2 weeks. Patients underwent the 100-ml water swallow test (WST) and FEES was evaluated using the following three scales: the Yale Pharyngeal Residue Severity Rating Scale, Penetration-Aspiration Scale, and the Dysphagia Outcome Severity Scale. An operative cohort of 19 patients (median age 75.8 years, 57.9% female) underwent an endoscopic operation on Zenker’s diverticulum, tight cricopharyngeal muscle diagnosed in videofluorography, or both. Patients completed the F-EAT-10 preoperatively and 3 months postoperatively. The cut-off score for controls was < 3 (sensitivity 94.0%, specificity 96.1%) suggesting that ≥ 3 is abnormal. Re-questionnaires for test–retest reliability analysis were available from 92 FEES patients and 123 controls. The intraclass correlation coefficient was excellent for the total F-EAT-10 score (0.93, 95% confidence interval 0.91–0.95). Pearson correlation coefficients were strong (*p* < 0.001) for each of the questions and the total score. Internal consistency as assessed by Cronbach’s alpha was excellent (0.95). Some correlations between findings in FEES and 100-ml WST with F-EAT-10 were observed. The change in subjective symptoms of operative patients paralleled the change in F-EAT-10. F-EAT-10 is a reliable, valid, and symptom-specific patient-reported outcome measure for assessing dysphagia among Finnish speakers.

## Introduction

Difficulty in swallowing, or dysphagia, is a common symptom related to many diseases. Dysphagia affects approximately 20% of the general population, 33% of individuals > 80 years living independently, and half of hospitalized patients [1–4]. Many neurological diseases affect swallowing and up to 80% of patients with acute stroke suffer from dysphagia [5]. In addition, esophageal and functional dysphagia, when the patient has subjective swallowing problems, but the swallowing is normal, usually affect young- and middle-aged adults. As dysphagia is also often unreported by patients and unrecognized and neglected by clinicians, the true prevalence of dysphagia is likely even higher than reported [6]. Dysphagia can lead to dehydration, malnutrition, social isolation, mental problems, pneumonia, and even death [6, 7]. In addition to its personal burden, dysphagia also leads to substantial healthcare costs [8].

Patient history and clinical examination are of utmost importance in dysphagia diagnostics. Dysphagia can be classified as either oropharyngeal or esophageal; a careful patient interview helps to identify the anatomical site and likely cause of dysphagia in most cases. In patients with oropharyngeal dysphagia, safety and efficiency of swallowing should be evaluated. Instrumental examinations, such as videofluorography or fiberoptic endoscopic evaluation of swallowing (FEES), are often necessary. Diagnostic tools in esophageal dysphagia include transnasal esophagoscopy, esophagogastroscopy, high-resolution manometry, or multichannel intraluminal impedance and pH monitoring. However, overuse of instrumental examinations is neither necessary nor cost effective. In dysphagia diagnostics, validated screening tools should be used to evaluate symptom severity and treatment outcomes. Several patient-reported outcome measures for oropharyngeal dysphagia assessment are available [9]. Some questionnaires are more generic, such as the Swallowing Quality of Life Questionnaire (SWAL-QOL) [10] or the Dysphagia Handicap Index (DHI) [11], whereas some are more disease specific, such as the M.D. Anderson Dysphagia Inventory (MDADI), which was developed to evaluate dysphagia-related quality-of-life among patients with head and neck cancer [12]. Many of these questionnaires are very detailed, extensive, and time consuming and are thus too cumbersome for clinical practice.

In 2008, Belafsky et al. developed the Eating Assessment Tool (EAT-10) to provide examiners with a rapid and easily scored dysphagia instrument that can be used at each patient visit to assess severity of dysphagia symptoms, quality of life, and treatment efficacy [13]. EAT-10 consists of 10 items related to the main aspects of dysphagia. Each item is rated on a 5-point scale from no difficulty (0 points) to severe difficulty (4 points). The sum of points from all 10 items is calculated and a score > 2 points is considered suggestive of dysphagia [13]. EAT-10 is easy to use and takes less than 2 min to complete [13–18]. EAT-10 has been shown to be a valid clinical tool and has good internal consistency and test–retest reliability [13]. In Europe, EAT-10 has been translated and validated in Spanish [14], Italian [15], European Portuguese [16], Swedish [19], Turkish [17], Greek [20], Dutch [21], and French [22]. The aim of this study was to validate a Finnish version of EAT-10 (F-EAT-10) to determine the clinical need for such assessment in Finland among Finnish speakers. We also aimed to investigate if F-EAT-10 can predict certain findings (particularly aspiration) and if this tool is applicable in a follow-up to assess changes in dysphagia symptoms.

## Materials and Methods

### Translation of EAT-10 into Finnish

Written consent to translate and validate EAT-10 was requested from Peter Belafsky, who owns the copyright of the original EAT-10. We used the forward–backward method in translation according to Wild et al. [23]. A native Finnish-speaking professional translator performed the forward translation from English to Finnish. A back translation was performed by a native English-speaking professional translator. Two experienced laryngologists (L-MA and PJ) also independently translated EAT-10 into Finnish. No critical differences were found after comparison of the translations. The final F-EAT-10 was achieved after laryngologists (L-MA, PJ, PP, TK, TI) of the Helsinki University Hospital, Department of Otorhinolaryngology – Head and Neck Surgery (HUH ORL-HNS) discussed F-EAT-10 together and made minor changes to improve the Finnish language (Table 1). A pilot test of F-EAT-10 was performed by L-MA and PJ on 10 dysphagic outpatients from HUH ORL-HNS (median age 55.2 years, range 18–79, 60% male). After reviewing the patient’s comments, no changes to F-EAT-10 questionnaire were deemed necessary.

**Table 1:** Items from the Finnish version of EAT-10 (F-EAT-10)

Item	Original version	Finnish version
	To what extent are the following scenarios problematic for you?	Missä määrin seuraavat tilanteet ovat teille ongelmallisia?
1	My swallowing problem has caused me to lose weight	Nielemisvaivani on aiheuttanut minulle painon laskua
2	My swallowing problem interferes with my ability to go out for meals	Nielemisvaivani häiritsee sitä, voinko mennä ulos syömään
3	Swallowing liquids takes extra effort	Nesteiden nieleminen on työlästä
4	Swallowing solids takes extra effort	Kiinteän ruoan nieleminen on työlästä
5	Swallowing pills takes extra effort	Tablettien nieleminen on työlästä
6	Swallowing is painful	Nieleminen on kivuliasta
7	The pleasure of eating is affected by my swallowing	Nielemiseni vaikuttaa ruokailusta nauttimiseen
8	When I swallow food sticks in my throat	Niellessäni ruoka takertuu kurkkuuni
9	I cough when I eat	Yskin syödessäni
10	Swallowing is stressful	Nieleminen on stressaavaa

### Controls and Patients

#### Controls

Otological and audiological outpatients recruited from HUH ORL-HNS served as controls. Controls were aged > 18 and < 85 years and had no history of symptoms, examinations, or treatment related to dysphagia or dysphonia, no history of difficult xerostomia or difficult reflux symptoms, and no diagnosed neurological diseases (except migraine). Moreover, controls had no history of diagnosed head and neck cancer, upper gastrointestinal or upper respiratory tract malignancy, or surgical procedures in upper aerodigestive tract. However, history of adenotomy and tonsillectomy were acceptable if the operation was performed > 1 year previously and if postoperative recovery was uneventful. The nurse interviewed the controls regarding the inclusion criteria before entry into the study. All controls completed the F-EAT-10 and the nurse measured the time required to complete the questionnaire to obtain information on the feasibility of F-EAT-10. To evaluate the test–retest reliability, the controls were asked to complete F-EAT-10 again after 2 weeks. The controls received a text message as a reminder to complete and return the F-EAT-10.

#### Patients

The study consisted of the following two patient groups with dysphagia: FEES patients and those operated endoscopically due to Zenker’s diverticulum, tight cricopharyngeal muscle, or both (operative patients). Included patients were > 18 years, native Finnish speakers, without notable psychiatric or cognitive disease, and able to complete F-EAT-10 independently.

#### FEES Patients

Patients with suspected oropharyngeal dysphagia who underwent FEES were recruited between September 2018 to June 2020 from four university hospital otorhinolaryngological and phoniatric departments (Helsinki, Kuopio, Tampere, Turku), one secondary care hospital ear, nose, and throat (ENT) department (Vaasa Central Hospital), and one secondary care hospital phoniatric (Central Ostrobothnia Central Hospital) department. F-EAT-10 was completed before FEES and again after 2 weeks to assess test–retest reliability. A text message or a phone call served as a reminder to complete and return the re-questionnaire. Because of possible symptom change during the 2-week period, the re-questionnaire also included a question to assess if the patient’s symptoms were the same as 2 weeks earlier (0 = no symptoms, 1 = less symptoms, 2 = same symptoms, 3 = slightly more symptoms, 4 = much more symptoms). Moreover, this also allowed assessment of criterion validity of whether F-EAT-10 could show possible symptom changes of FEES patients during the follow-up.

### FEES Protocol and Water Swallow Test

In FEES, swallowing was evaluated with a transnasally passed thin, diameter of 2.6 or 3.4 mm, videoendoscope when the patient was given different textures and bolus volumes [24]. The bolus textures used were liquid (water), nectar (blueberry soup), semi-solid (puree), and solid (cookie). The examination started with small boluses (the tip of a teaspoon) and continued to larger ones (maximum tablespoon). The examination was modified according to patient swallowing ability. An experienced ENT specialist or a phoniatrician with a speech and language pathologist (SLP) or an experienced SLP alone performed and evaluated their own FEES tests using the following three scales: the Yale Pharyngeal Residue Severity Rating Scale [25], the Penetration-Aspiration Scale (PAS) [26], and the Dysphagia Outcome Severity Scale (DOSS) [27]. In addition, the following were evaluated: saliva retention in vallecula and pyriform sinuses, movements of lateral pharyngeal walls and base of the tongue, velopharyngeal closure, vocal fold closure, and sensory findings of arytenoids and tip of the epiglottis by touching with an endoscope. When evaluating the patient’s Yale Pharyngeal Residue Severity Rating Scale and PAS, the worst result was marked as the final result. The baseline F-EAT-10 score and FEES findings were compared to examine how the symptoms correlated with the findings (criterion validity). All FEES patients underwent the 100-ml water swallow test (WST) [28]. In the WST, the patient was asked to drink 100 ml water continuously at his or her own pace. The number of swallows needed, possible coughing during or after the examination, and the patient’s wet-hoarse voice after drinking were assessed [28]. The test was considered passed if the patient was able to drink continuously without coughing with less than nine swallows and with no voice change. The cause of dysphagia was assessed afterwards in all FEES patients.

### Operative Patients

The operative patients group included dysphagic patients undergoing endoscopic operation because of Zenker’s diverticulum, tight cricopharyngeal muscle diagnosed in videofluorography, or both. The operative patients were recruited between September 2018 and October 2020 from four university hospital otorhinolaryngological clinics (Helsinki, Kuopio, Oulu, and Turku). The operations included stapler-assisted diverticuloesophagostomy for Zenker’s diverticulum, carbon dioxide (CO_2_) laser cricopharyngeal myotomy, cricopharyngeal dilatation with balloon, or rigid hypopharyngoscopy combined with possible botulinum toxin injection to the muscle. Patients who underwent an operation to cricopharyngeal muscle frequently, for example, cricopharyngeal dilatation due to sequelae of head and neck cancer were excluded. The patients completed F-EAT-10 preoperatively and 3 months postoperatively. The questionnaire also included an additional question regarding possible symptom changes due to operation. This question evaluated whether the change in F-EAT-10 was parallel to subjective symptom change (criterion validity) and was scored from 0–4 (0 = no symptoms, 1 = less symptoms, 2 = same symptoms, 3 = slightly more symptoms, 4 = much more symptoms than before operation).

### Questionnaire Acceptance

Questionnaires with missing answers were not accepted. If the participant selected two adjacent numbers for the same question in F-EAT-10, the mean was calculated and recorded. However, if the participant answered one question with two non-adjacent numbers, the answer was rejected. Only participants with a total F-EAT-10 score in the first questionnaire were included in the study. Participants whose re-questionnaire’s total F-EAT-10 score was impossible to calculate were not excluded from the study but their answers in the deficient re-questionnaire were not accepted.

### Ethical Considerations

The participants were given both oral and written information about the study protocol and provided written consent before study entry. The Ethics Committee of the Helsinki and Uusimaa Hospital District approved the study protocol. A research permission was applied in each hospital. This study was conducted in accordance with the Declaration of Helsinki (The World Medical Association 2013).

### Statistical Analysis

The minimum sample size requirement was 100 participants for both FEES patients and controls according to a subject to item ratio of 10:1 [29, 30]. Allowing for a 10% drop-out rate, the required sample size was increased to 111 participants.

F-EAT-10 scores were reported as means (standard deviation, SD), as this would allow a better description of differences in scores and is also more comparable to other validation studies. Other results (e.g., age) are reported descriptively as frequencies, medians (range), or means (SD) according to the data distribution.

Data from controls and FEES patients were used for internal consistency and reliability analysis. Cronbach’s alpha was used to assess the internal consistency of the F-EAT-10 baseline questionnaire for controls and FEES patients together and for FEES patients alone. For Cronbach’s alpha, values ≥ 0.7 but < 0.8 were considered to show acceptable consistency, values > 0.8 but < 0.9 good consistency, and values ≥ 0.9 excellent consistency [31].

The following two methods were used to assess test–retest reliability in FEES patients and controls together and in FEES patients alone: by correlating each question and the total scores in baseline tests and re-tests with a Pearson correlation coefficient and with the intraclass correlation coefficient (ICC). ICC with 95% confidence intervals (CI) was assessed with a two-way mixed-effect model based on single ratings and absolute agreement. Interpretation was as follows: < 0.50, poor; between 0.50 and 0.75, fair; between 0.75 and 0.90, good; > 0.90, excellent [32]. Pearson correlation coefficient was used to allow comparison of results with other validation studies. A Pearson correlation < 0.3 was considered weak, between 0.3 and 0.49 moderate, and ≥ 0.5 strong [33].

The difference in median age between FEES patients and controls was compared with the Mann–Whitney *U* test and the difference in gender distribution with chi-square test. The correlation between age and F-EAT-10 scores was assessed with Spearman’s rho. The Mann–Whitney *U* test was used to compare the scores of each of the F-EAT-10 questions and the total scores between FEES patients, operative patients, and controls. Based on the data distribution, Spearman’s rho was used to assess the correlation between the baseline F-EAT-10 total score and the scores for PAS (liquid, nectar, puree, cookie), Yale Pharyngeal Residue Rating Scale (vallecula, pyriform sinus), DOSS, and status findings (saliva retention in vallecula and pyriform sinuses, movements of the lateral pharyngeal walls and the base of the tongue, velopharyngeal closure, vocal fold closure, and sensory testing). Comparisons of the baseline F-EAT-10 total score to findings from FEES patients were assessed with Mann–Whitney *U* test or Kruskal–Wallis test. Kruskal–Wallis and Mann–Whitney *U* tests were used for comparisons of the baseline F-EAT-10 total score to aspiration and penetration in PAS and if the patient passed the 100-ml WST, respectively. To determine which F-EAT-10 score would indicate aspiration or penetration in FEES, we used a receiver operating characteristic (ROC) curve with Youden Index, which indicates the maximum potential effectiveness of a biomarker. The subjective symptom changes in FEES patients and operative patients were compared to the change of the total F-EAT-10 score using the Kruskal–Wallis test. The change in the total F-EAT-10 score in operative patients was determined using Wilcoxon signed-rank test. An experienced statistician was consulted regarding the statistical analysis.

All statistical analyses were performed with the IBM SPSS Statistics for Windows (Version 26.0; IBM Corp., Armonk, NY, USA). *P* values less than 0.05 were considered statistically significant.

## Results

### Normative Data

The median age of the 180 controls was 57.0 years (range 18.3–82.1); 62.2% was female (Table 2). All 180 F-EAT-10 questionnaires at baseline and the returned re-questionnaires were acceptable. The median time needed to complete the questionnaire was 30 s (range 10–120). The mean total score of the baseline F-EAT-10 was 0.47 (SD 0.96, range 0–6). According to the original article of Belafsky, the upper limit of the normal cohort was calculated mean + 2SD. For F-EAT-10 this was 2.39, suggesting that the cut-off score for F-EAT-10 is < 3 (sensitivity 94.0%, specificity 96.1%). The total score was 0 in 129 (71.7%), 1 in 32 (17.8%), and 2 in 12 (6.7%) controls. Only 7 controls (3.9%) scored between 3 and 6. The re-questionnaire was received from 123 controls (68.3%) and the mean F-EAT-10 total score was 0.36 (range 0–4). These 123 re-questionnaires were used in the test–retest reliability analysis. A minor decrease was observed in the total score of the re-questionnaire compared with the baseline scores. The median time to complete the re-questionnaire was 14 days (range 6–36).

**Table 2:** Characteristics of patients and controls and mean F-EAT-10 total scores

Group	Age (y)	*n* (%)	Female (%)	F-EAT-10 (SD) range
Controls	≤ 20	8 (4.4)	62.5	1.1 (1.5) 0–4
	21–40	46 (25.6)	58.7	0.4 (0.8) 0–4
	41–60	51 (28.3)	64.7	0.6 (1.1) 0–6
	61–79	73 (40.6)	61.6	0.3 (0.7) 0–4
	≥ 80	2 (1.1)	100.0	2.0 (2.8) 0–4
	Total	180 (100)	62.2	0.5 (1.0) 0–6
FEES patients	≤ 20	3 (2.6)	66.7	13.0 (7.2) 5–19
	21–40	12 (10.3)	75.0	14.2 (8.4) 2–28
	41–60	29 (24.8)	55.2	14.8 (8.0) 0–36
	61–79	61 (52.1)	44.3	16.2 (9.3) 0–37
	≥ 80	12 (10.3)	66.7	17.1 (12.1) 3.5–38
	Total	117 (100)	53.0	15.7 (9.1) 0–38
Operative patients	41–60	1 (5.3)	0.0	21.0 (-)
	61–79	11 (57.9)	72.7	22.7 (7.6) 11–35
	≥ 80	7 (36.8)	42.9	20.6 (4.2) 15–27
	Total	19 (100)	57.9	21.8 (6.3) 11–35

*F-EAT-10:* Finnish Eating Assessment Tool, *FEES:* fiberoptic endoscopic evaluating of swallowing, *SD:* standard deviation

### FEES Patients

A total of 127 FEES patients were screened for the study. Nine FEES patients (7.1%) were excluded from the analysis because their total scores for the baseline F-EAT-10 could not be calculated, and one patient was excluded due to age (17 years). In the final analysis, there were 117 FEES patients with median age 69.7 years (range 19.5–90.4), of which 53.0% was female (Table 2). The baseline F-EAT-10 total scores and FEES findings of these 117 patients were evaluated in criterion validity analysis. The re-questionnaire was received from 97 FEES patients (82.9%), of which 50.5% was female. Re-questionnaires from five FEES patients (5.2%) were not accepted as the total F-EAT-10 scores could not be calculated. These patients were not otherwise excluded from the analysis. Thus, we had 92 re-questionnaires from FEES patients for the test–retest reliability analysis. The mean total score of these re-questionnaires was 14.4 (SD 9.6, range 0–38). The most common causes for dysphagia among all 117 FEES patients were functional (*n* = 31, 26.5%), head and neck or esophageal malignancies (*n* = 16, 13.7%), and neurological (*n* = 15, 12.8%). All FEES patients were eating or drinking orally at least to some extent. The main etiology of dysphagia for FEES patients and the mean total F-EAT-10 scores in different diagnostic groups are presented in Table 3. Some patients have undergone FEES to examine their oropharyngeal dysphagia symptoms or the safety of their swallowing although their main problem may have been esophageal.

**Table 3:** Etiology of dysphagia in FEES patients and correlations with mean F-EAT-10 total scores

Etiology of dysphagia	*n* (%)	F-EAT-10 (SD) range
Functional*	31 (26.5)	16.2 (9.7) 0–36
Head and neck or esophageal cancer	16 (13.7)	22.3 (6.6) 14–37
Neurological	15 (12.8)	17.4 (9.9) 0–32
Esophageal (reflux, motility disorders, esophagitis)	14 (12.0)	14.5 (9.1) 4–38
Presbyphagia	12 (10.3)	13.8 (7.9) 2–25
Dry mouth/throat	9 (7.7)	13.2 (5.0) 7–21
Sensation of dysphagia but swallowing is normal	9 (7.7)	6.2 (5.0) 0–17
Compression (goiter, osteophyte, surgical material after ACIF operation)	3 (2.6)	13.3 (11.0) 1–22
Cricopharyngeal problem or Zenker’s diverticulum	4 (3.4)	20.5 (8.2) 10–30
Other specific reason (EDS, DM, OM, scleroderma)	4 (3.4)	12.0 (9.5) 5–25

*ACIF:* anterior cervical interbody fusion, *DM:* dermatomyositis, *EDS:* Ehlers-Danlos syndrome, *F-EAT-10:* Finnish Eating Assessment Tool, *FEES*: fiberoptic endoscopic evaluating of swallowing, *OM*: overlap myositis, *SD*: standard deviation

^*^Patient has subjective swallowing problems, but the swallowing is normal

### Clinical Validity

There were no differences in gender distribution between controls and FEES patients. While FEES patients were older than controls (*p* < 0.001), age was not significantly correlated to the baseline F-EAT-10 total score in controls (r = -0.10, *p* = 0.17) or FEES patients (r = 0.11, *p* = 0.24). However, in question 3 (swallowing liquids), there was a correlation with age and a higher score in FEES patients (r = 0.28, *p* = 0.002). FEES patients had higher total scores and higher individual question scores than controls; all differences were statistically significant (*p* < 0.001) (Table 2).

### Internal Consistency and Reliability

The internal consistency of the F-EAT-10 total score as assessed by Cronbach’s alpha was excellent (0.95) for FEES patients and controls together and good (0.88) for FEES patients alone.

The ICC for determining the test–retest reliability was excellent for the F-EAT-10 total score of FEES patients and controls together (0.93, 95% CI 0.91–0.95). ICC was good for FEES patients alone (0.84, 95% CI 0.76–0.89). Pearson correlation coefficients were statistically significant (*p* < 0.001) for each of the questions and in the baseline F-EAT-10 total scores for FEES patients and for FEES patients and controls together. Pearson correlations were strong in FEES patients and controls together for the baseline F-EAT-10 total score and in each single question. Moreover, Pearson correlations were strong in FEES patients alone for the baseline F-EAT-10 total score and for the single questions. All ICCs and Pearson correlations are presented in Table 4**.**

**Table 4:** Test–retest reliability in FEES patients and controls

F-EAT-10 question	ICC for FEES patients (95% CI), *n* = 117	Pearson correlations for FEES patients, *n* = 117	ICC for FEES patients and controls (95% CI), *n* = 297	Pearson correlation for FEES patients and controls, *n* = 297
1. Weight loss	0.76 (0.66–0.84)	0.76 *	0.81 (0.75–0.85)	0.81*
2. Ability to go out for meals	0.79 (0.68–0.86)	0.81*	0.87 (0.83–0.90)	0.88*
3. Swallowing liquids disorder	0.67 (0.54–0.77)	0.68*	0.76 (0.69–0.81)	0.76*
4. Swallowing solids disorder	0.69 (0.56–0.78)	0.69*	0.87 (0.83–0.90)	0.87*
5. Swallowing pills disorder	0.70 (0.58–0.79)	0.71*	0.83 (0.79–0.87)	0.83*
6. Painful swallowing	0.65 (0.52–0.76)	0.65*	0.72 (0.64–0.78)	0.72*
7. Reduced pleasure of eating	0.70 (0.58–0.79)	0.70*	0.84 (0.80–0.88)	0.84*
8. Food sticks in the throat	0.68 (0.47–0.80)	0.72*	0.87 (0.82–0.90)	0.88*
9. Cough during eating	0.79 (0.69–0.85)	0.79*	0.86 (0.82–0.89)	0.86*
10. Stressful swallowing	0.68 (0.56–0.78)	0.69*	0.83 (0.78–0.87)	0.83*
Total score	0.84 (0.76–0.89)	0.84*	0.93 (0.91–0.95)	0.93*

*CI*: confidence interval, *F-EAT-10*: Finnish Eating Assessment Tool, *FEES*: fiberoptic endoscopic evaluating of swallowing, *ICC*: intraclass correlation coefficient

^*^p < 0.001

### Criterion Validity

For criterion validity assessment, findings from 117 FEES patients were compared to their baseline F-EAT-10 total score. These correlations between PAS (liquid, nectar, puree, cookie) and Yale Pharyngeal Residue Rating Scale (vallecula, pyriform sinus) are presented in Tables 5 and 6, respectively. In DOSS, a negative correlation with the baseline F-EAT-10 total score was observed (r = -0.39, *p* < 0.001), indicating that patients with normal diet (DOSS 7) had the lowest F-EAT-10 total score (Table 7). Based on the ROC curve using Youden Index (data not shown), the F-EAT-10 cut-off score was ≥ 22 (sensitivity 54.5%, specificity 19.2%) for aspiration of liquid in PAS and ≥ 16 (sensitivity 70.4%, specificity 42.0%) for penetration or aspiration. The cut-off score was ≥ 22 (sensitivity 57.9%, specificity 17.5%) for penetration or aspiration for nectar, ≥ 16 (sensitivity 68.4%, specificity 45.4%) for puree, and 16 (sensitivity 61.9%, specificity 42.5%) for cookie.

**Table 5:** Correlation of mean F-EAT-10 total scores to Penetration-Aspiration Scale in FEES patients

Consistency	PAS finding**	*n* (%)	F-EAT-10 (SD) range	F-EAT-10 correlation coefficient (Spearman’s rho)	*p* value
Liquid, *n* = 115	normal	88 (76.5)	14.6 (8.3) 0–36	0.17	0.075
	penetration	16 (13.9)	17.5 (11.2) 0–38		
	aspiration	11 (9.6)	18.5 (9.0) 1–29		
	penetration or aspiration	27 (23.5)	18.0 (10.2) 0–38		
Nectar, *n* = 116	normal	97 (83.6)	14.7 (8.7) 0–38	0.27	0.004
	penetration	16 (13.8)	21.3 (7.4) 7–34		
	aspiration	3 (2.6)	20.3 (18.1) 1–37		
	penetration or aspiration	19 (16.4)	21.2 (9.1) 1–37		
Puree, *n* = 116	normal	97 (83.6)	15.0 (9.1) 0–38	0.15	0.106
	penetration	19 (16.4)	17.8 (7.4) 5–29		
	aspiration	0 (0)	-		
Cookie, *n* = 108	normal	87 (80.6)	14.5 (8.6) 0–38	0.10	0.323
	penetration	21 (19.4)	15.9 (8.0) 1–29		
	aspiration	0 (0)	-		

*F-EAT-10*: Finnish Eating Assessment Tool, *FEES*: fiberoptic endoscopic evaluating of swallowing, *PAS*: Penetration-Aspiration Scale, *SD*: standard deviation

^**^According to the Penetration-Aspiration Scale: normal = PAS 1, penetration = PAS 2–5, aspiration = PAS 6–8

**Table 6:** Residue findings according to the Yale Pharyngeal Residue Severity Rating Scale and correlations with mean F-EAT-10 total scores

Location of Residue	Consistency	Residue severity***	*n* (%)	F-EAT-10 (SD) range	Spearman’s correlation	*p* value
Vallecula	Liquid	none	77 (67)	14.2 (8.8) 0–36	**0.21**	**0.023**
		trace	24 (21)	16.6 (7.1) 1–28		
		mild	13 (11)	19.0 (11.4) 0–38		
		moderate	1 (1)	29 (-) -		
		severe	0 (0)	-		
	Nectar	none	70 (60)	14.9 (8.9) 0–36	**0.15**	**0.112**
		trace	29 (25)	15.7 (8.2) 1–38		
		mild	14 (12)	19.1 (9.9) 0–34		
		moderate	2 (2)	14.0 (12.7) 5–23		
		severe	1 (1)	37 (-) -		
	Puree	none	47 (41)	13.7 (8.5) 0–36	**0.21**	**0.027**
		trace	32 (28)	15.5 (9.3) 0–36		
		mild	21 (18)	16.5 (6.6) 5–27		
		moderate	13 (11)	19.1 (10.7) 0–38		
		severe	3 (3)	20.7 (14.6) 5–34		
	Cookie	none	42 (39)	14.5 (8.3) 0–36	**0.10**	**0.290**
		trace	21 (20)	11.4 (7.8) 0–25		
		mild	26 (24)	17.0 (8.3) 1–36		
		moderate	14 (13)	16.5 (10.1) 0–38		
		severe	4 (4)	14.8 (6.7) 5–20		
Pyriform	Liquid	none	81 (70)	14.1 (8.7) 0–36	**0.22**	**0.020**
sinus		trace	20 (17)	17.8 (7.8) 7–32		
		mild	12 (10)	18.9 (10.7) 0–38		
		moderate	1 (1)	14 (-) -		
		severe	1 (1)	29 (-) -		
	Nectar	none	77 (66)	14.5 (8.8) 0–36	**0.18**	**0.050**
		trace	25 (22)	18.4 (8.3) 5–38		
		mild	8 (7)	13.5 (6.7) 5–24		
		moderate	5 (4)	21.0 (13.3) 0–34		
		severe	1 (1)	37 (-) -		
	Puree	none	66 (57)	13.8 (8.3) 0–36	**0.24**	**0.011**
		trace	21 (18)	15.6 (9.6) 0–35		
		mild	19 (16)	20.3 (8.4) 6–38		
		moderate	9 (8)	15.8 (9.5) 0–28		
		severe	1 (1)	29 (-) -		
	Cookie	none	62 (58)	13.3 (8.5) 0–36	**0.23**	**0.018**
		trace	22 (21)	16.1 (8.7) 1–38		
		mild	12 (11)	17.6 (7.0) 5–28		
		moderate	10 (9)	17.3 (9.4) 0–29		
		severe	1 (1)	16 (-) -		

*F-EAT-10*: Finnish Eating Assessment Tool, *SD*: standard deviation

^***^According to the Yale Pharyngeal Residue Severity Rating Scale

**Table 7:** Dysphagia Outcome Severity Scale ratings and correlations with mean F-EAT-10 total scores in FEES patients

DOSS rating	*n* (%)	F-EAT-10 (SD) range	F-EAT-10 Correlation coefficient (Spearman’s rho)
Total	117	15.7 (9.1) 0–38	-0.39*
Level 7: Normal in all situations	43 (36.8)	11.9 (9.1) 0–38	
Level 6: Within functional limits	35 (29.9)	15.3 (8.7) 0–36	
Level 5: Mild dysphagia	21 (17.9)	19.1 (8.6) 1–38	
Level 4: Mild-moderate dysphagia	12 (10.3)	18.4 (11.0) 0–36	
Level 3: Moderate dysphagia	4 (3.4)	25.8 (6.1) 18–32	
Level 2: Moderately severe dysphagia	1 (0.9)	23 (-) -	
Level 1: Severe dysphagia	1 (0.9)	37 (-) -	

*DOSS*: Dysphagia Outcome Severity Scale, *F-EAT-10*: Finnish Eating Assessment Tool, *FEES*: fiberoptic endoscopic evaluating of swallowing, *SD*: standard deviation

^*^p < 0.001

FEES patients who passed (*n* = 76) the 100-ml WST had a mean baseline F-EAT-10 total score 14.0 (SD 8.4), whereas those who did not pass the test (*n* = 27) had a mean score of 18.2 (SD 9.3). The difference between groups was statistically significant (*p* = 0.04). Moreover, those who coughed (*n* = 15) during the 100-ml WST had a mean baseline F-EAT-10 total score of 23 (SD 8.0) and those who did not cough (*n* = 88) had a mean total score of 13.8 (SD 8.3). This difference was statistically significant (*p* = 0.001). In addition, the F-EAT-10 total score tended to increase if the patient coughed after the 100-ml WST or if the patient’s voice became wet-hoarse, although these differences were not statistically significant (*p* = 0.08, and *p* = 0.32, respectively).

In status findings, there was a positive correlation with saliva retention in vallecula and pyriform sinuses and baseline F-EAT-10 total scores (r = 0.27, and r = 0.29, respectively, *p* < 0.001). A statistically significant difference was also observed between normal and abnormal movements of the lateral pharyngeal walls and F-EAT-10 total scores (*p* = 0.02). However, no statistically significant differences were noted between the F-EAT-10 total score and normal versus abnormal findings in velopharyngeal closure, movement of the base of the tongue, vocal fold closure, or sensory testing on the tip of the epiglottis or on the arytenoids.

Of the 92 FEES patients who returned re-questionnaire, 73 (53.4% female) also answered the question regarding possible symptom changes during the 2-week follow-up. Most of the FEES patients felt their symptoms were unchanged (*n* = 42, 57.5%) or felt a slight symptom improvement (*n* = 20, 27.4%). Only a few patients considered their symptoms as slightly worse (*n* = 6, 8.2%) or absent (*n* = 5, 6.8%). The median F-EAT-10 total score change decreased in those who experienced less symptoms (-0.5), the same symptoms (-0.5), or were asymptomatic (-5.0), but increased among those who experienced more symptoms (0.5). However, this result was not statistically significant (*p* = 0.61).

### Operative Patients

Of the 22 operative patients, the re-questionnaire was received from 21 patients (95.5%). Of these 19 answered every question, including the question concerning possible symptom changes after the operation. The data from these 19 patients (median age 75.8 years, range 56.4–87.1, 57.9% female) were used in criterion validity analysis. The characteristics of operative patients and mean F-EAT-10 total scores are presented in Table 2. Cricopharyngeal balloon dilatation was performed on nine patients, of which one also had botulinum toxin injection, seven patients had stapler-assisted operation of Zenker’s diverticulum, two patients had cricopharyngeal myotomy performed with CO_2_ laser, and one patient underwent cricopharyngeal dilatation with rigid hypopharyngoscopy combined with botulinum toxin injection. The mean F-EAT-10 score was 21.8 (SD 6.3, range 11–35) at baseline and 11.4 (SD 10.0, range 0–31) after a 3-month follow-up and the change in scores was statistically significant (*p* < 0.001). Most of the patients felt that they were asymptomatic (*n* = 10, 52.6%) or had less symptoms (*n* = 8, 42.1%) than before operation. One patient (5.3%) felt that her symptoms were unchanged. No patients experienced more symptoms. The median F-EAT-10 total score decreased in those who experienced less symptoms (-7.0) or were asymptomatic (-17.5). The F-EAT-10 score did not change in the patient who felt her symptoms were unchanged. However, possible due to small number of operative patients, this result was not statistically significant (*p* = 0.31).

## Discussion

Our results indicate that F-EAT-10 is a valid patient-reported outcome measure for dysphagia. This study showed a significant difference in total scores between controls and dysphagia patients and in each of the 10 questions, thus indicating the validity of F-EAT-10 as a screening tool. Most of the controls had scores of 0 in F-EAT-10 and mean score was 0.47, which is consistent with the previous validation studies [13–16, 18, 19, 22]. The cut-off score for our controls was determined < 3 points suggesting that ≥ 3 is abnormal, consistent with the original article of Belafsky and in many other validation projects [13, 15, 16, 19]. However, there are some EAT-10 studies where the cut-off score was 2 [18, 34]. Although a few of our controls scored > 2 points, they all reported having no deglutition problems during an interview by the nurse. However, one or more questions in F-EAT-10 reminded them of some previous problems they recorded, but this was not a reason for exclusion from the study. However, it would be useful to have a time interval in EAT-10 (e.g., asking about symptoms in the previous month) in which the subject evaluates his or her symptoms of deglutition.

There were no missing values in the F-EAT-10 questionnaires from controls, which indicates that the questions were easy to answer for asymptomatic subjects. Moreover, the median time to complete F-EAT-10 for controls was only 30 s, suggesting that F-EAT-10 is feasible as demonstrated in other studies [13–18]. However, some baseline questionnaires (7.1%) and re-questionnaires (5.2%) from FEES patients were not answered correctly and thus the total scores were not countable. This result is similar to that of the S-EAT-10 validation project (7%) [19]. In general, missing answers were rare and, differed between questions, indicating that there are no specific questions that are difficult to answer. In some studies, participants without proper reading skills or with a severe neurological disease or dementia have been excluded [19, 22]. This is consistent with our exclusion criteria, in which participants unable to complete the questionnaire independently or with any notable psychiatric or cognitive disease are not eligible. Our observation suggests that F-EAT-10 is easy to complete among our study population. Re-questionnaires were received more often from FEES patients (*n* = 97, 82.9%) and operative patients (*n* = 21, 95.5%) than from controls (*n* = 123, 68.3%). All participants received a text message or a phone call as a reminder, but we assume that asymptomatic subjects were less motivated to complete and return the re-questionnaires than patients.

The Cronbach’s alpha value (0.95) was excellent for the total F-EAT-10 score in controls and FEES patients together and was also good for FEES patients alone (0.84), indicating an excellent to good internal consistency. According to the previous validations, Cronbach’s alpha for the total EAT-10 score varied from 0.87 (Sp-EAT-10) to 0.96 (the original EAT-10 of Belafsky), suggesting that our result is consistent with these previous studies [13–19, 22, 35]. The ICC indicating test–retest reliability was 0.93 (0.91–0.95) for both controls and FEES patients together and 0.84 (0.76–0.89) for FEES patients alone, indicating excellent to good reproducibility. In the original article of EAT-10, ICC ranged from 0.72 to 0.91 and the highest ICCs were approximately 0.90 for the total score [18, 19]. Nevertheless, the I-EAT-10 total score reached ICCs as high as 0.95 (patients) and 0.98 (controls). In FEES patients and controls together, Pearson correlations were strong for the total score (0.93) and in every single question (0.72–0.88), consistent with the previous studies [15–17, 19, 22, 36]. In addition, Pearson correlations were strong for the total score (0.84) and every single question (0.65–0.81) for FEES patients alone. Our FEES patients completed the re-questionnaire after a 2-week follow-up. As the FEES procedure is usually followed by instructions to help deglutition and because the patient’s dysphagia symptoms may fluctuate, we asked about possible symptom change in the re-questionnaire. Although some patients reported symptom changes, some FEES patients did not answer this question and, we cannot exclude the possibility that their symptoms might also have changed. This is a probable explanation for why our test–retest results did not reach as high ICCs and correlations as observed in other studies [15, 18, 35].

The mean F-EAT-10 total score was different between diagnostic groups. Patients without significant findings in FEES had the lowest total scores and those with a neurological cause or a malignancy had the highest total scores. Patients with dry mucous membranes/xerostomia, or presbyphagia, had elevated, but not very high total scores. Thus, F-EAT-10 scores were as expected in different diagnostic groups.

For the criterion validity assessment, we prospectively collected numerous findings in FEES to compare with the F-EAT-10 total score. While we found some statistically significant correlations in PAS and the Yale Pharyngeal Residue Rating Scale, the correlations were mainly weak. In DOSS, negative and moderate correlations were evident, indicating that F-EAT-10 total scores increased with poorer swallowing. Moreover, some positive correlations were found in the status findings. Our results were similar to those of the Hebrew EAT-10 (EAT-10_Heb_) validation study, where weak correlations with EAT-10 scores and pathological findings in FEES were found [37]. Additionally, in the I-EAT-10 validation, weak correlations with EAT-10 total score and DOSS and with PAS in semisolids were observed [15]. In a recently published study, mild-to-moderate correlations were found between EAT-10 and PAS scores depending on the patient’s diagnosis. Higher EAT-10 scores were also significantly correlated with higher PAS scores. However, these patients underwent videofluoroscopy [38]. Thus, we conclude that objective findings in FEES may also correlate with subjective patient symptoms, although the correlations were mainly weak in our study.

We also wanted to study whether the F-EAT-10 total score can predict aspiration risk. As the causes for dysphagia were usually benign in our FEES patients (e.g., functional, presbyphagia, dry mouth, or throat) or esophageal, there were only a few patients who aspirated. As no FEES patients aspirated with puree or cookie and only three with nectar, the cut-off scores were mainly determined for penetration (PAS > 1) and thus included possible aspiration. An F-EAT-10 total score of ≥ 16 was the cut-off score for penetration (PAS > 1) with liquid, puree, or cookie; the corresponding score was ≥ 22 with nectar (ROC curves). Due to the small number of FEES patients with PAS > 1, the sensitivity and specificity were not usually high. Moreover, we used the Youden Index, which works best if there are approximately the same number of patients with and without the finding. Although some previous studies have concluded that EAT-10 scores may predict aspiration [7, 18, 22, 38–40], there are also contrary results [41]. The heterogeneity of the patient groups and study settings also makes comparisons across studies challenging. Belafsky’s group observed aspiration (PAS > 5) in videofluoroscopic swallowing studies and concluded that a cut-off score of 16 reached a sensitivity of 71% and specificity of 53%. However, their patient group was heterogenous and included patients with esophageal dysphagia [39]. Taken together, there is no consensus of the cut-off score for aspiration and further studies are needed.

WST is a rapid screening tool for dysphagia [28, 42]. Thus, we also studied the results of the 100-ml WST of FEES patients and correlations to F-EAT-10 total score. In FEES patients who passed the test, a statistically significant difference was observed in F-EAT-10 total score compared with those who did not pass. Moreover, a statistically significant difference in F-EAT-10 total score was observed among those who did not cough during the test compared with those who did cough. Our result suggests that the 100-ml WST along with F-EAT-10 may predict swallowing problems, as demonstrated in recently published studies [43, 44].

We aimed to test whether F-EAT-10 can capture possible subjective symptom changes during a 2-week (FEES patients) or a 3-month (operative patients) follow-up period, indicating another aspect of criterion-based validity. The F-EAT-10 total score decreased in those FEES patients who experienced less symptoms, the same symptoms, or were asymptomatic and increased among those who experienced more symptoms. However, the result was not statistically significant, possibly because of the small number of FEES patients in the two subgroups (asymptomatic and slightly more symptoms). F-EAT-10 scores captured symptom changes in operative patients in most cases. Thus, F-EAT-10 can also be used to evaluate patient symptoms after an endoscopic operation on Zenker’s diverticulum, tight cricopharyngeal muscle diagnosed in videofluorography, or both.

There are some limitations to the present study. Some questionnaires were excluded because of missing or uninterpretable answers. Among operative patients, subjective symptom changes in some patients did not parallel changes in the F-EAT-10 score and the number of operative patients was low. Evaluation of subjective symptom changes after operative treatment might be more accurate if asked after a shorter time than 3 months. This study was a multicenter study, as the need to validate a Finnish dysphagia outcome tool was nationwide. However, only a few FEES patients were recruited in some hospitals and most patients and all controls were from the Helsinki University Hospital. There were 12 professionals who performed FEES, which may influence the interpretation of FEES findings. On the other hand, F-EAT-10 performed well despite 12 colleagues participating in the study. Moreover, well-known classifications were used, which should make the results more congruent.

## Conclusions

We validated F-EAT-10 in asymptomatic controls and in FEES patients with different dysphagia etiologies and in dysphagia patients who underwent an endoscopic procedure. Our results indicate that F-EAT-10 is a valid instrument to evaluate deglutition problems and is also applicable for follow-up. F-EAT-10 can be helpful in identifying patients who are at risk for penetration and aspiration. In addition, there are some correlations between patients’ subjective symptoms measured by F-EAT-10 and objective findings in FEES.
